# Facing Hymenoptera Venom Allergy: From Natural to Recombinant Allergens

**DOI:** 10.3390/toxins7072551

**Published:** 2015-07-09

**Authors:** Amilcar Perez-Riverol, Débora Lais Justo-Jacomini, Ricardo de Lima Zollner, Márcia Regina Brochetto-Braga

**Affiliations:** 1 Laboratório de Biologia Molecular de Artrópodes-LBMA-IB-RC-UNESP (Univ Estadual Paulista), Av. 24-A, n_ 1515, Bela Vista, Rio Claro 13506-900, SP, Brazil; E-Mails: aperezriverol@gmail.com (A.P.-R.); dmjacomini@gmail.com (D.L.J.-J.); 2 Laboratório de Imunologia e Alergia Experimental-LIAE, Departamento de Clínica Médica, Faculdade de Ciências Médicas, FCM, Universidade Estadual de Campinas-UNICAMP, Rua Tessália Vieira de Camargo n_ 126, Cidade Universitária “Zeferino Vaz”, Campinas 13083-887, SP, Brazil; E-Mail: zollner@unicamp.br; 3 Centro de Estudos de Venenos e Animais Peçonhentos-CEVAP (Univ Estadual Paulista), Rua José Barbosa de Barros, 1780, Fazenda Experimental Lageado, Botucatu 18610-307, SP, Brazil

**Keywords:** Hymenoptera venom, allergy, recombinant allergens, “omics” approaches, diagnosis, immunotherapy

## Abstract

Along with food and drug allergic reactions, a Hymenoptera insect Sting (Apoidea, Vespidae, Formicidae) is one of the most common causes of anaphylaxis worldwide. Diagnoses of Hymenoptera venom allergy (HVA) and specific immunotherapy (SIT) have been based on the use of crude venom extracts. However, the incidence of cross-reactivity and low levels of sensibility during diagnosis, as well as the occurrence of nonspecific sensitization and undesired side effects during SIT, encourage the search for novel allergenic materials. Recombinant allergens are an interesting approach to improve allergy diagnosis and SIT because they circumvent major problems associated with the use of crude venom. Production of recombinant allergens depends on the profound molecular characterization of the natural counterpart by combining some “omics” approaches with high-throughput screening techniques and the selection of an appropriate system for heterologous expression. To date, several clinically relevant allergens and novel venom toxins have been identified, cloned and characterized, enabling a better understanding of the whole allergenic and envenoming processes. Here, we review recent findings on identification, molecular characterization and recombinant expression of Hymenoptera venom allergens and on the evaluation of these heterologous proteins as valuable tools for tackling remaining pitfalls on HVA diagnosis and immunotherapy.

## 1. Hymenoptera Venom Allergy: General Epidemiological Considerations

Approximately 20,000 species of Hymenoptera from Apoidea (bees) and Formicidae (ants) together with 15,000 species of Vespoidea (wasps, yellow jackets) have been identified as clinically relevant [1]. Stinging events caused by these species account for 1.5%–34.1% of all types of anaphylaxis reported per year [2]. Hymenoptera venom can cause local and systemic allergic reactions including anaphylaxis. Several factors, such as the insect type, concomitant cardiovascular and respiratory disease, age, mastocytosis, high levels of serum tryptase, and previous use of angiotensin-converting enzyme (ACE) inhibitors, determine the severity of the clinical symptoms [2]. Systemic reactions occur mainly in allergic patients, compromising the proper functioning of the respiratory and circulatory systems. Clinical symptoms include generalized urticaria, angioedema, blood pressure fall, bronco-spasms, cardiac and respiratory arrest, and anaphylactic shock [3]. The estimated prevalence of systemic reactions associated with Hymenoptera venom allergy (HVA) is 0.3%–8.9%, while anaphylaxis occurs in 0.3%–42.8% of the cases [4]. Life-threatening cases occur in 0.1%−0.5% of inhabitants in Europe and the United States [5] and are mainly due to systemic hypersensitive reactions mediated by the specific allergen IgE, which binds to the Fc receptors on the surface of several cell types, including mastocytes and basophiles. The recognition of the allergen-IgE immuno complex by Fc receptors induces degranulation and release of inflammation mediators such as histamine, leukotrienes, and several cytokines [6].

To date, collected data about the incidence of Hymenoptera stings in humans have not been consistently documented. However, an estimated 56% to 94% of adults worldwide have been stung at least once in their lifetime [7]. Insect venom anaphylaxis incidence is highly influenced by geographic and economic backgrounds. An epidemiologic study developed on a pediatric population of Ireland [8] showed that 37.5% of children suffered at least one sting in their lifetime, while a proportion of 56.3% was observed in a similar survey conducted in Israel [9]. As mentioned by the authors, variations in geographic situation, weather factors, and flora/fauna composition between those countries may have caused the differences in the reported results.

Several studies showed that accident events are less frequent in the pediatric population than in adults [10]. Similarly, rates of insect sting hypersensitivity have been reported as lower in children (0.4%–8%) than in adults (3%) [11]. On the other hand, greater percentages of sting events are reported for the male compared to the female population [12], with beekeepers showing the highest incidence rate.

Unfortunately, little is known about the incidence of Hymenoptera venom allergy in low-income countries, where most cases are not well documented, leading to an underestimation of their impact on human health and quality of life. Most of the survey studies currently reported were conducted in developing countries where prevalence of HVA is assumed to be low, given the small percentage of people who live or develop their economic activities near populations of Hymenoptera insects.

## 2. Hymenoptera Venom Composition: “Omics” Approaches

Molecular characterization of Hymenoptera venom represents a milestone in the design and development of novel, safe and more efficient diagnosis and therapy strategies. Hymenoptera venom is a complex mixture of low molecular weight compounds such as biogenic amines, lipids and carbohydrates, cationic peptides, and high molecular weight proteins that represent the so-called allergens [13]. Usually, low molecular weight components are responsible for local toxic reactions, while systemic reactions are due to allergenic proteins. To date, hundreds of Hymenoptera venom allergens have been identified and characterized. Most of them are enzymes such as hyaluronidase, phospholipase A1 and A2, antigen 5, serine proteases, and acid phosphatases [5,14].

The application of high-throughput techniques along with several omics approaches are revealing novel, clinically relevant allergens and venom toxins that could be useful in order to understand the whole “venome” [14] and, subsequently, the allergenic and envenoming processes. Heterologous expression of these molecules, especially in the case of venom allergens, will help in improving component-resolved diagnosis (CRD) of venom allergy and specific immunotherapy (SIT). Here, we focus our analysis on Hymenoptera venom allergenic proteins recently discovered and characterized using different omics approaches and in some cases, high throughput screening techniques that, in conjunction, greatly contribute to their production as recombinant molecules.

### 2.1. Exploring Hymenoptera Venom Proteome

Most of the Hymenoptera venom allergens currently cloned and recombinantly expressed have been characterized using proteomic approaches, including the usual SDS-PAGE, 2D protein electrophoresis, mass spectrometry, and chromatographic techniques. To date, honeybee venom (HBV) and yellow jacket venom (YJV) remain the most explored among any other from the Hymenoptera species, mainly due to their high clinical and epidemiological impacts. Several pioneer works using chromatographic and immunochemical techniques led to the discovery of four major allergens from HBV: phospholipase A2 (*Api m* 1), hyaluronidase (*Api m* 2), acid phosphatase (*Api m* 3) and melittin (*Api m* 4) [15,16,17]. Meanwhile, early proteomic, biochemical, and immunological works with wasp venom enabled the identification of the three major allergens in *Vespula vulgaris* venom: phospholipase A1 (*Ves v* 1), hyaluronidase (*Ves v* 2), and antigen 5 (*Ves v* 5) [18]. Commercial recombinant forms of *Api m* 1, *Ves v* 5 and *Ves v* 1 are now available and have been successfully used for differentiation of true double sensitization from cross-reactivity cases during diagnosis [19,20]. Nevertheless, despite being widely used in allergy diagnosis, reports of low sensitivity levels of IgE detection when *Api m* 1 is used as the unique marker for HBV allergy [21] have prompted the search for novel *A. mellifera* venom allergens that could help in overcoming this inconvenience.

Peiren *et al.* identified three novel proteins by mapping the *A. mellifera* venom proteome using 2D gel electrophoresis and mass spectrometry (MALDI TOF/TOF-MS and Q-TRAP LC-MS/MS) techniques [22]. Full gene codifying for the novel protein 2, later named as icarapin (*Api m* 10), was subsequently amplified by 5′-Rapid amplification of cDNA ends (5′-RACE), cloned and expressed using a bacterial commercial system [23]. As *Api m* 10 is a low-abundance protein in HBV, a recombinant (r) form of the allergen was evaluated for the sIgE reactivity against a panel of allergy patients’ serum [24]. r*Api m* 10 reacted with 80% of patients’ sera, showing that it represents a novel allergen that is unrepresented in honeybee crude venom extracts but that could be used to improve specific allergy diagnosis by increasing the sensitivity rates. In fact, it was recently shown that r*Api m* 10 expressed in insect cells reacted with 61.8% of the sera from patients allergic to HBV [25]. Peiren *et al.* also identified an *A. mellifera* venom component similar to the major royal jelly protein 8 (MRJP 8) [22]. Further efforts to clone and express this MRJP 8 (*Api m* 11.0101) and a similar protein MRJP 9 (*Api m* 11.0201) in insect cells led to the identification of both molecules as minor allergens of *A. mellifera* venom [26].

The glycosylated *Api m* 11.0101 protein expressed on High Five *Tricolupsia ni* insect cells reacted with 53.2% of the allergic patients’ sera, while the recombinant allergen expressed in *Spodoptera frugiperda* Sf9 cells, lacking α 1,3-core fucose (cross-reactive carbohydrate determinants (CCD)-free), reacted with 14.9%. Moreover, MRJP9 expressed in High Five cells reacted with 61.7% of the patients’ sera, while CCD-devoid recombinant allergen recognized 34% of them. Higher values of allergen sIgE reactivity with MRJP9 produced in High Five cells reflected the fact that this isoallergen has only three *N*-glycosylations, which is half of that of MRJP 8. Given these results, authors proposed that *Api m* 11.0101 and *Api m* 11.0201 are minor HBV isoallergens with IgE-sensitizing potential that could be contributing to improve the efficacy of CRD of HBV allergy by helping to identify the subsets of patients sensitized to these proteins. Furthermore, this result may also contribute to tackling the royal jelly-related allergic reactions (asthma, dermatitis and anaphylaxis) in individuals using cosmetics, food supplements, and commercial medical products derived from royal jelly (RJ) [27,28].

Allergens B and C, now *Api m* 3 and *Api m* 5, respectively, were initially identified in the high molecular fraction of *A. mellifera* venom [29] by combining chromatographic, proteomic and immunologic assays. Early attempts to clone *Api m* 3 failed to obtain a full-length gene sequence [30]. By combining an *in silico* analysis of *A. mellifera* venom peptides and available genome sequences, Grunwald *et al.* successfully designed PCR primers for the full gene amplification of *Api m* 3 [31] and its subsequent expression using insect cells. In that study, r*Api m* 3 showed reactivity with 37% of the sera from the HBV-sensitized patients analyzed. The sequencing of the purified natural *Api m* 5 using tandem mass spectrometry followed by an *in silico* comparative analysis with the *A. mellifera* genome also allowed its cloning and proper expression in insect cells [32]. Interestingly, by using a similar approach, authors were able to identify an acid phosphatase from *Vespula vulgaris* venom (*Ves v* 3) having a high percentage of identity with antigen 5 from HBV. *Api m* 3 and *Api m* 5 were also evaluated on CRD of HBV allergy [25] and reacted with 50% and 58.1% of the sera of bee venom allergic patients, respectively.

In a recent work, Blank *et al.* reported the identification of two novel vitellogenins in honeybee (*Api m* 12) and *Vespula vulgaris* (*Ves v* 6) venoms, also using tandem mass spectrometry-based protein sequencing [33]. Recombinant forms of both allergens expressed in insect cells are glycosylated but CCD-free, as no reaction was detected with anti-HRP (horseradish peroxidase) rabbit serum, specific for α 1,3-core fucosylation of N-glycans. This feature prevents the occurrence of cross-reactivity, transforming *Api m* 12 and *Ves v* 6 as interesting alternatives for improving the diagnosis of HBV and YJV allergy.

For decades, classical proteomic approaches using 2D electrophoresis and mass spectrometry have uncovered around 30 defined compounds in *A. mellifera* venom, including allergenic proteins, low molecular mass peptides, and biogenic amides. In contrast, Van Vaerenbergh *et al.* recently identified in a single study 83 new molecules in HBV, including 17 putative toxins, by using a combinatorial peptide ligand library approach followed by shotgun LC-FT-ICR MS analysis [34]. In a similar study, also using a combinatorial peptide ligand and nano-LC/MALDITOF/TOF-MS system, four novel proteins in HBV were described [35]. These works reinforced the relevance of applied high-throughput techniques and novel proteomics methods in order to elucidate the whole proteome composition of Hymenoptera venoms as well as identify novel low-abundance allergenic molecules that could be used in panels of defined allergens for CRD.

Similar to studies with HBV, several proteomic approaches have also been used to determine the composition of venom from different wasp species. A classical bottom-up procedure using 2D protein electrophoresis and tandem mass spectrometry was used to elucidate *Polybia paulista* venom proteome in an attempt to understand the envenoming process [36]. In this work, authors describe the presence of more than 84 proteins in the venom of this Brazilian endemic wasp, most of them for the first time. In this regard, the proteomic characterization of a hyaluronidase from *P. paulista* venom (*Poly p* 2) [37] has allowed its cloning, sequencing, 3D-structural modeling, and recombinant expression using an *E. coli* system [38,39]. The authors demonstrated that the recombinant form reacts with sera from patients allergic to *P. paulista* venom in the same way as the natural allergen [39]. A similar approach is being used to obtain the recombinant forms of the two other major allergens—phospholipase A1 (*Poly p* 1) and antigen 5 (*Poly p* 5)—from the venom of this species as proteomic analysis and characterization of the referred proteins are already available [40,41,42]. In fact, data derived from proteomic characterization of *Poly p* 5 were used to identify linear B-cell epitopes in the structure of this allergen [43]. Using 66 peptides covering the full-length protein, authors were able to identify nine linear B-cell epitopes reacting with human IgG, one of which (peptide 7) also reacts with human IgE. Further analysis combining structural modeling of *Poly p* 5 and the peptide sequences revealed the presence of a critical fragment of peptide 7 (WAKTKE). Authors suggested that this sequence is settled at a loop on the protein surface representing the major B-cell binding epitope. Considering these findings, they proposed that it could be used as a candidate for peptide immunotherapy or production of modified recombinant Ag 5 (r*Poly p* 5), having reduced allergenicity and an improved safety and tolerance induction profile, a major goal for SIT success [43].

### 2.2. Genomic and Transcriptomic Approaches

Genomic approaches have also been used to clone and express genes from Hymenoptera venom. In this regard, the sequencing of the *A. mellifera* genome [44] represented a milestone for the identification of novel Hymenoptera genes, including those encoding venom allergens. Annotated gene sequences obtained from *A. mellifera* have been used in comparative analysis of the transcriptome-derived data from other Hymenoptera species. For instance, analysis of expressed sequence tags (ESTs) derived from the *Bombus ignitus* venom gland has recently allowed full-length gene amplification and sequencing of venom phospholipase 2 from this species [45].

As noted, relatively little information is available regarding gene and protein sequences from venom allergens of endemic species from the Southern Hemisphere. Consequently, few studies have been published on recombinant expression of venom allergens from Hymenoptera species of this geographic region. The integration of transcriptomic, peptidomic, and proteomic approaches will provide large amounts of data that may be used to reduce the lack of commercial recombinant allergens from typical species of this region and thus improve specific diagnosis of allergy. Interestingly, a recent study of allergic rhinitis in subtropical regions that combined transcriptomic, proteomic, and allergenomic approaches to grass pollen enabled the detection of more than 17,000 transcripts, several corresponding to putative allergenic proteins [46]. In addition, at least four novel allergens, namely *Sor h* 1, *Sor h* 13, *Sor h* 2, and *Sor h* 23, were identified in this study.

Transcriptomics has emerged as a common strategy for exploring venom composition and understanding the envenoming process. The use of this approach provides a large amount of biological data, helping to unravel the protein and peptide composition of venoms. A transcriptome analysis of the venom gland from *Tetramorium bicarinatum*, a widely distributed ant, led to the identification and sequencing of 364 high-quality ESTs, 18% corresponding to venom compounds, 62% of which match previously annotated allergens sequences [47]. Several identified ESTs match putative pilosulin-like peptides, the major allergens described in *Myrmecia pilosula* and also the main cause of HVA in Australia [48]. Another important finding was the detection of transcripts with *Sol i* 3-like peptide sequence, a major allergen from the *Solenopsis invicta* ant, which is a *Ves v* 5 3D homolog. A similar transcriptomic analysis was conducted using venom glands of *Dinoponera quadriceps*, a giant ant from the Neotropical region [49]. A cDNA library of the venom gland was constructed and analyzed using the Sanger sequencing method and deep whole-transcriptome shotgun sequencing. From 420 independent clones derived from the cDNA library, 15% of contigs and singlets match previously identified venom toxins, while 5% represent hypothetical proteins. Deep RNA sequencing reveals 3807 contigs that were divided in 23 functional groups according to the Gene Ontology and Cluster of Orthologous Groups (COG) database. For both procedures, sequences matching annotated toxins and venom components were namely dinoponeratoxins, venom allergens, phospholipase-like toxin peptides and lethal-like proteins. Within venom allergens, sequences matching those encoding *Solenopsis invicta* phospholipase A1 (*Sol i* 1) and antigen 5 (*Sol i* 3) were found at high rates. Interestingly, 65% of the transcripts that were obtained using deep RNA sequencing procedure did not match any known sequence, showing the potential of this approach to identify novel venom compounds.

Transcriptomic coupled with proteomic analyses of venom glands have also been carried out to elucidate the molecular basis of envenoming processes after an accident with poisonous animal species including snake [50,51,52,53,54], jellyfish [55], spider [56,57] and scorpion [58]. These studies have resulted in the identification of several new venom toxins, allowing a greater understanding of the envenoming process.

Although venom composition of clinically relevant Hymenoptera species of the Northern Hemisphere has been elucidated, novel molecules are still being described mainly by using novel high-throughput screening assays combined with several omics approaches. This fact shows the potential of applying these strategies in the identification and characterization of novel venom toxins and allergens that could be used in the pharmaceutical and biotechnology industries, particularly for improvement of diagnosis and treatment of venom allergy.

## 3. Hymenoptera Recombinant Allergens and Diagnostic Tests

Specific diagnosis of HVA represents a prerequisite step for the success and safety profile of immunotherapy procedures. For decades, the use of crude venom extracts predominated in the design of diagnostic strategies. However, high levels of cross-reactivity occur during diagnosis using crude extracts as allergenic material [59]. Homologous proteins in the venom of two different groups or species of insects may share specific IgE (sIgE) epitopes and/or can have CCDs. The latter accounted for up to 60%–70% of the patients’ sera that cross-reacted with venom of different Hymenoptera species [60,61]. Cross-reactive occurrence prevents the proper identification of culprit venom leading to inclusion of non-relevant allergens on specific therapy schemes, compromising the safety profile of therapy intervention as nonspecific sensitization. Undesired adverse side effects can occur during SIT.

Diagnosis is based on patient history concerning allergic reactions, positive skin test, and *in vitro* tests that include allergen-specific IgE detection and basophil activation test (BAT) [62,63,64]. Double positive is a common issue during Hymenoptera venom allergy diagnosis using crude venom extracts, since up to 59% of the patients show reaction to both HBV and YJV [59]. These rates have been explained by occurrence of true double sensitization or cross-reactivity, the latter accounting for 70%–80% of double sensitization in the case of these two species [65].

Component-resolved diagnosis based on the use of defined, properly characterized and purified recombinant allergens has emerged as a powerful tool for circumventing problems in venom allergy diagnosis [66,67]. Commercial recombinant forms of *Api m* 1 from HBV and *Ves v* 5, *Ves v* 1 from *V. vulgaris* venom are now available and have been successfully used for differentiation of true double sensitization from cross-reactivity cases [19,20] during diagnosis. Studies performed mainly with non-glycosylated variants of these recombinant allergens have provided the proper identification of the culprit venom. In brief, by using non-glycosylated recombinant forms of *Api m* 1*, Api m* 2, and *Ves v* 5, expressed in *E. coli*, it was possible to distinguish bee and/or wasp sensitization in 29 allergic patients previously diagnosed with double positivity when venom extracts were used as the allergenic material [68]. A similar study, performed using r*Ves v* 5 and r*Api m* 1 and sera from 20 patients previously diagnosed as positive for sIgE presence for both bee and wasp venom, showed a rate of double positivity/double negativity/single positivity for r*Api m* 1 and r*Ves v 5* of 12/1/9 patients on sIgE test. Thereafter, a double-positivity/double-negativity/single-positivity ratio of 6/2/14 was obtained in the case of BAT [19]. Müller *et al.* found that only 47% of 76 patients previously identified as double positive for whole venom extract from honeybee and wasp reacted with r*Ves v 1*, r*Ves v 5*, and *rApi m 1* during sIgE detection [69]. In addition, 100% of the patients previously detected as wasp venom single positive reacted to r*Ves v* 1 and/or r*Ves v* 5, and 78.3% of single HBV allergic patients had sIgE for r*Api m* 1. Recombinant forms of *Ves v* 1 and *Ves v* 5 expressed on insect cells were able to identify 16/20 patients previously identified as double positive on sIgE detection assays [65]. Recently, natural and recombinant forms of antigen 5 and phospholipase A1 from *Vespula vulgaris* and *Polistes dominulus* (*Pol d* 1 and *Pol d* 5) proved to be necessary for proper identification of culprit venom during diagnosis of allergy to the venom of these species [70].

As seen, detection of sIgE to r*Ves v* 1, r*Ves v* 5, and r*Api m* 1 represents a very reliable strategy in order to recognize double sensitization from cross-reactivity during diagnosis of honeybee and/or YJV allergy. However, it is important to note that *Api m* 1 has a limited diagnostic sensitivity (60.0% to 72.2%), restricting its usefulness for sIgE detection as a unique relevant allergen included during HBV allergy diagnosis [21]. As an alternative, it was proposed that the inclusion of other honeybee allergens in panels for CRD might assist in solving the problem related to the low sensibility. In fact, by using a set of recombinant *A. mellifera* venom allergens (*Api m* 1, *Api m* 2, *Api m* 3, *Api m* 4, *Api m* 5, *Api m* 10), it was possible to detect sIgE reactivity for at least one allergenic protein in 94.4% of the patients’ sera [25]. Therefore, the inclusion of other recombinant forms of HBV allergens that differ from r*Api m* 1, and which are now available, may improve the results of venom allergy diagnosis by raising the levels of sIgE detection. Interestingly, to date, more than 60 Hymenoptera allergenic proteins have been identified, cloned, and recombinantly expressed [14], and most of them could be exploited as candidates to be used in clinical practices for improving venom allergy diagnosis.

## 4. Venom Immunotherapy: Trends and Prospects for the Use of Recombinant Allergens

There are two major approaches in order to treat systemic allergic reactions caused by Hymenoptera stings. Acute management mainly comprises the use of self-injectable epinephrine, while long-term management is related to SIT intervention, which is the only disease-specific approach currently used. It involves the administration of increasing amounts of crude venom extract, typically beginning with 0.1–1.0 µg, in order to induce tolerance in allergic patients. SIT has proved to be highly effective. In fact, for wasp venom allergy, more than 90%–95% of the patients did not show any systemic reactions after being re-stung, while more than 80% did not develop reactions in the case of honeybee [4,10,71]. However, local and severe adverse reactions may occur during treatment, with higher rates observed for patients receiving bee venom compared to wasp venom [72,73]. It has been reported that 20%−40% of the patients suffered systemic side effects during SIT with HBV, while 5%−10% suffered in the case of wasp venoms [72]. In addition, in a recent systemic review, Boyle *et al.* published an interesting meta-analysis of one quasi-randomized controlled and six randomized trials of Hymenoptera venom SIT [74]. Trials included ant, wasp, and bee immunotherapy in children and adults, using a sublingual (1) or subcutaneous (6) route, accounting for a total of 392 participants. Meta-analysis showed that only 3/113 (2.7%) participants receiving SIT experienced a subsequent systemic allergic reaction to a sting challenge, compared with 37/93 (39.8%) of the untreated participants (risk ratio (RR) 0.10, 95% confidence interval (CI) 0.03 to 0.28). It is important to note that no differences were identified between patient groups or modes of treatment, showing that the sublingual could be considered a feasible administration route for SIT, which could also improve patients’ compliance since it is also a less invasive route for allergen administration. Authors also found a significant risk of systemic adverse reaction outcomes. In summary, the meta-analysis showed that SIT using venom extracts is a highly effective strategy to treat Hymenoptera venom allergy, but it is related to a significant risk of severe adverse reaction occurrence. Therefore, novel strategies are required in order to reduce the incidence of severe adverse reactions, thus improving the SIT safety profile.

To date, immunotherapy schedules remain highly heterogeneous as they vary in the nature of the allergenic material used, dose amount, administration route and the time necessary to reach the protective and maintenance dose. For the latter, protocols could be divided into *slow protocols*, requiring several weeks to attain protective and maintenance doses, *rush* (also *semi-rush*) *protocols* requiring several days (4–7 days), and finally, *ultra-rush* protocols requiring only hours to 1–2 days [75]. In general, it has been stated that *slow*, *semi-rush*, and *rush* protocols are safer than *ultra-rush*. A comparative study between *ultra-rush* and *semi-rush* buildup with jack jumper ant (*Myrmecia pilosula*) venom found high percentages of occurrence of side effects in patients included on an *ultra-rush* schedule [76]. However, some authors have reported similar rates of adverse reactions during immunotherapy using *ultra-rush*, *rush*, and *slow* schedules [77,78]. Then, the safety profile of strategies, with respect to the time used to reach the protective or maintenance doses, probably relies more on the type of insect causing the allergy than on the procedure used to induce tolerance.

Subcutaneous is the main administration route currently used on SIT procedures for HVA. However, different approaches, such as painless routes for allergen application, are needed in order to improve patient compliance. Sublingual immunotherapy (SLIT) has been successfully used on allergic rhinitis treatment and provides an interesting, alternative method of allergen administration for venom allergy immunotherapy [79,80]. More than 60 randomized double-blind, placebo-controlled trials performed with SLIT have demonstrated its efficacy and safety profile for the treatment of different allergic diseases [81]. To date, only one study has evaluated the effect of SLIT on Hymenoptera venom allergy. A proof-of-concept clinical trial was conducted in humans using HBV and patients with a history of large local reactions (LLRs) after an insect sting [82]. Significant reduction in the diameter of LLRs (20.5 to 8.5 cm) was observed for the active patients, while no effects were detected in the placebo group. However, further studies are required in order to establish the efficacy and safety of SLIT as a more compliant and suitable procedure to treat HVA.

To date, mainly crude and commercially standardized venom extracts are used as allergenic materials in SIT. However, as noted, their uses are associated with the occurrence of nonspecific sensitization and severe adverse reactions during SIT [74], with higher effects when the culprit insect is not properly identified. It is important to point out that crude venom extracts present variations on allergenic compound composition and stability, leading to considerable variations in the outcomes of SIT. Recombinant allergenic proteins emerge as an interesting option in order to face these disadvantages [67].

Thus far, recombinant allergens from Hymenoptera venom have been tested mainly for diagnosis improvement. In fact, to our knowledge, only one study has evaluated the *in vivo* induction of tolerance to insect venom during SIT by using a recombinant Hymenoptera allergen [83]. In their work, the authors developed a murine model of *Vespula vulgaris* venom allergy and found lower rates of toxic effects during immunotherapy with the recombinant form of *Ves v* 5 than with the crude venom extract. Authors also found high levels of tolerance induction when the recombinant allergen was used as allergenic material.

To date, no study has been conducted in humans in order to evaluate recombinant allergens as an alternative material to be use in Hymenoptera venom immunotherapy. However, results obtained with recombinant allergens for other allergic diseases [84,85] show the potential of applying a similar approach on HVA specific treatment. In theory, heterologous expression could lead to the development of personalized panels of safer unmodified or modified recombinant allergens that may reduce the incidence of severe adverse reactions and nonspecific sensitization during Hymenoptera venom SIT. However, it would be necessary to reduce economic costs and improve the safety and efficacy profile in order to position this technology as a competitive commercial alternative to the use of crude venom extracts, for the development of a novel commercially licensed product.

## 5. Production of Recombinant Allergens: From Gene to Proper Expression Systems

As previously discussed, recombinant DNA technology represents a powerful tool to overcome the remaining pitfalls in the diagnosis and immunotherapy of Hymenoptera venoms. Large amounts of standardized, highly purified, non-modified recombinant allergens with a similar physiochemical and immunologic profile as the natural variants can be produced by using this technology. Modified hypoallergenic and chimeric proteins can also be obtained in order to improve the efficacy and safety of SIT. In this regard, recombinant allergens engineered to have a reduced specific IgE reactivity while conserving T-cell epitopes have been successfully tested, as they induce tolerance without occurrence of undesirable side effects [86,87]. All these features are moving the scenario of recombinant allergens for some allergic diseases towards the development of CRD and future patient-tailored therapy schemes [88].

Future progress in the scenario of recombinant Hymenoptera venom allergen production highly depends on the selection of a proper system for its heterologous expression. As noted, more than 60 Hymenoptera venom allergenic proteins have been cloned and expressed using bacteria, yeast, and the mammalian cell system [14]. *E. coli* remains a highly feasible system for allergenic protein expression, as it enables the production of non-glycosylated molecules aiding to solve CCD-mediated cross-reactivity [68], therefore allowing the proper identification of the culprit venom. However, the use of the prokaryotic system for allergen expression is compromised in several cases, since most Hymenoptera allergens currently identified have conformational IgE epitopes and carbohydrate motifs. Proper folding and glycosylation steps are crucial for heterologous production of molecules with a physiochemical and immunologic profile similar to the natural variants. Attempts to express Hymenoptera allergens using the *E. coli* system have rendered different results in terms of structural and immunological similarities of heterologous proteins in comparison with the natural counterpart. A recombinant non-glycosylated form of *Api m* 1 expressed in *E. coli* showed an identical structural and immunological profile in relation to the natural enzyme, indicating a native-like folding of the protein [89]. In that case, glycosylation was not critical for the allergenicity of the molecule. In contrast, heterologous expression of *Api m* 2 using *E. coli* yielded a protein with lower enzymatic activity and sIgE binding capacity compared to those of the natural (n) form of *Api m* 2 and r*Api m* 2 expressed in baculovirus-infected insect cells [90]. Interestingly, Skov *et al.* reported that a recombinant form of *Vespula vulgaris* hyaluronidase (*Ves v* 2) obtained by using the *E. coli* system had similar levels of enzymatic activity as n*Ves v* 2 after *in vitro* refolding and purification steps [91]. The purified r*Ves v* 2 was crystallized and analyzed using sequence alignment and structural comparison, showing a high level of 3D structure homology with natural *Api m* 2 from HBV. In addition to this, Justo Jacomini *et al.* reported that the recombinant hyaluronidase obtained from the venom of the endemic Brazilian wasp *P. paulista* (*Poly p* 2) using the *E. coli* system was recognized by allergic patient sera at higher levels than n*Poly p* 2 [39]. In this case, heterologous protein was expressed in an insoluble form, and was then solubilized but not re-natured. The study proposed that allergen recognition was mediated by linear rather than conformational B-cell epitopes previously described on n*Pol p* 2 by combining proteomic procedures [37,38] and bioinformatics tools. Hyaluronidase variants from venom of queen and worker individuals of *Solenopsis invicta* (*Sol i* 2) have been successfully cloned and expressed in *E. coli* [92]. High levels of soluble r*Sol i* 2 was produced at 16 °C, which was recognized by human sIgE from allergic patients’ sera. The authors pointed out that, by using *E. coli*, an inexpensive and time-saving protocol for functional allergen production was developed, which is a common objective when heterologous protein expression procedures are designed. As observed, heterologous expression using *E. coli* remains a feasible strategy to obtain large amounts of functional allergens that could be used in diagnosis and treatment of HVA. However, it is important to highlight that optimal expression conditions should be explored for each allergen, and that, in several cases, bacteria may not be used for heterologous expression due to the inability to achieve the proper protein folding.

Limitations associated with recombinant expression of Hymenoptera venom allergens in prokaryotes could be tackled by using eukaryotic expression systems currently available, namely yeast, baculovirus-infected insect cells, plants and mammalian cells. Recently, *Spodoptera frugiperda* Sf9 insect cells have received particular interest because high levels of N-glycosylated lacking α 1,3-core fucosylation allergens, properly folded and displaying all sIgE epitopes profile, could be obtained using this system [14,65]. Commercial forms of r*Api m* 1 (ImmunoCAP i208; Thermo Fisher Scientific, Uppsala, Sweden), r*Ves* v 1, and r*Ves v* 5 produced in baculovirus-infected insect cell line Sf9 are now available for specific diagnosis of *A. mellifera* or/and *V. vulgaris* venom allergy [20,63,93]. n*Ves v* 1 and n*Ves v* 5 are non-glycosylated proteins that share non-sIgE epitopes with n*Api m* 1; therefore, recombinant variants of these allergens have been successfully used to identify culprit venom in individuals previously diagnosed with double sensitization. However, some concerns have emerged when using this system for expression of allergens that are glycosylated in their natural form, as recombinant, non-fucosylated (CCD-free) allergens obtained in this system have, in general, lower diagnostic sensitivity than those displayed by natural allergens or crude venom extracts. As noted above, r*Api m* 1 expressed in Sf9 cells lacking α-1,3-linked fucose and N-linked glycosylation site was described as a low-sensitivity recombinant variant for HBV allergy diagnosis [21,94]. A study conducted by Jakob *et al.* showed that this variant was able to detect 72% of patients diagnosed with HBV allergy [95], which is similar to previous reports for other r*Api m* 1 variants, but lower than levels obtained in diagnosis with n*Api m* 1 (80%). Further analysis showed that those sensitivity differences are mainly associated with CCD recognition on natural material by sIgE in patients’ sera. Even when the authors concluded that benefits associated with avoidance of cross-reactivity and false double sensitization justified the use of this non-glycosylated recombinant form of r*Api m* 1 during diagnosis, these results support the idea that novel strategies are required in order to increase the sensitivity of diagnosis when using this r*Api m* 1 variant. One of these strategies is the use in CRD of recombinant forms of recently identified HBV allergens (*i.e.*, r*Api m* 10, r*Api m* 5, r*Api m* 6) that are under-represented in crude venom extracts, but are still recognized by sIgE in allergic patients. In summary, considering structural and immunological features, the insect cells, especially Sf9, represent a highly efficient system for heterologous expression of Hymenoptera allergens, as they ensure proper protein folding and avoidance of cross-reactivity by producing α-1,3-linked fucose-lacking molecules.

The methylotrophic yeast *Pichia pastoris* is a system widely used for expression of recombinant proteins, including several allergens. Features like high yields, genetic simplicity, capability to perform post-translational modifications, and the ability to ensure proper protein folding makes it an attractive system for protein production in the biotechnological and pharmaceutical industries. To date, however, a limited number of Hymenoptera venom allergens have been expressed using this yeast. For instance, high levels of a non-enzymatically active form of *Ves v* 1 were produced in *P. pastoris* [96] after introducing a point mutation at the active site of the allergen. The modified r*Ves v* 1 form was produced as a secreted soluble protein facilitating the subsequent purification steps. The allergens recognized sIgE from allergic patients, inhibited binding of sIgE to crude venom extract, and induced *in vitro* histamine release in sensitive basophils. Similarly, a recombinant form of the hypoallergenic antigen 5 from *Polybia scutellaris—*a South American wasp—was obtained using the *P. pastoris* system [97]. The allergen was engineered to avoid non-native glycosylation and was expressed at high levels (27 mg/L) in a scale-up schedule for optimal methanol concentration determination. Comparative immunological assays showed a similar IgE epitope profile of the recombinant molecule in relation to the natural variant, showing its potential as a candidate for the improvement of component-resolved diagnosis of *P. scutellaris* venom allergy. Considering these results and general features related to protein expression, *P. pastoris* appears as an inexpensive and suitable option for immunologically active allergen production, especially for research and academic use.

Determination of optimal expression conditions, and rational design of molecules to avoid cross-reactivity incidence and assure high yields of the active protein production, remain important issues to tackle in order to improve allergen expression using this system.

To our knowledge, few or no works have been published for heterologous production of Hymenoptera venom allergens using plant and mammalian cell-based systems. However, in the case of plant cells, this situation could change in the next few years, given the increasing interest in developing “*molecular farming*” as a cost-effective alternative for production of pharmaceutical proteins. Plant cell-based systems are easy to scale up for large amounts of low-cost biomass production, having lower risk of protein contamination with human pathogens compared to bacteria, yeast, or mammalian cells [98]. In addition, plant cells are able to perform full post-translational modifications in the expressed molecule, ensuring proper folding and biological activity [99]. Meanwhile, although 50%–80% of the recombinant biopharmaceuticals approved each year are produced in mammalian cells, no commercially available Hymenoptera venom allergens have been obtained using this system. Expression of heterologous protein using mammalian cells is expensive, and the required post-translational modifications for proper allergen folding can be achieved using more cost-effective systems such as yeast and insects cells.

The success of CRD relies on the proper selection of protocols and systems used for recombinant allergen expression. In turn, the selection of the expression system depends highly on the structural and immunological features of each allergen. To date, the bacteria *E. coli* remains the simplest and most inexpensive system for heterologous protein production, being commonly used for academic and research purposes. In the case of Hymenoptera venom allergens, the use of *E. coli* also helps to avoid the presence of CCDs, as no glycosylation is produced in these cells, therefore decreasing the incidence of cross-reactivity. However, this feature can also limit its use due to the inability to perform post-translational modifications or to allow the formation of disulfide bonds, often leading to improper protein folding and loss of conformational IgE epitopes. Thus, a refined balance among proper folding, conservation of IgE conformational epitopes, and avoidance of CCDs is required in order to preserve allergenicity and prevent the incidence of cross-reactivity. In this context, Hymenoptera allergens engineered to express as non-glycosylated, CCD-free proteins in insect cells have proven to be a highly efficient alternative and are now commercially available.

## 6. Concluding Remarks

Recent progress on identification and molecular characterization of Hymenoptera venom allergens enables the production of modified and non-modified recombinant allergens. By using different “omics” branches allied high-throughput screening techniques, we are now exploring whole “venome” [14], generating a large amount of biological data and hence detecting novel allergens that can be used for designing more specific and accurate venom allergy diagnosis strategies. The inclusion of novel, clinically relevant, commonly non-glycosylated and highly purified allergens in component-resolved diagnosis (CRD) proved to drastically reduce cross-reactivity during allergy diagnosis. Consequently, a significant improvement of specific immunotherapy (SIT) has been achieved. Furthermore, the ability to produce homogeneous panels of allergens with a defined physiochemical profile enhances the possibilities of their application in SIT schemes and, even more so, in the development of a personalized therapy. In theory, this approach could lead to a decrease in the incidence of severe systemic side effects during treatment. However, several challenges remain to be addressed in order to license the use of recombinant allergens for therapeutic interventions. Moreover, the production of hypoallergenic recombinant variants, or T-cell epitope peptides, along with the search for a less invasive administration route, could improve the therapy’s safety profile, the patient’s quality of life and the success in facing Hymenoptera venom allergy (HVA).
